# Self-organization of porous anodic alumina films studied *in situ* by grazing-incidence transmission small-angle X-ray scattering†

**DOI:** 10.1039/c8ra02913j

**Published:** 2018-05-23

**Authors:** Jonas Evertsson, Nikolay A. Vinogradov, Gary S. Harlow, Francesco Carlà, Sarah R. McKibbin, Lisa Rullik, Weronica Linpé, Roberto Felici, Edvin Lundgren

**Affiliations:** Division of Synchrotron Radiation Research, Lund University SE-22100 Lund Sweden jonas.evertsson@sljus.lu.se; ESRF – The European Synchrotron 71 Avenue des Martyrs 38000 Grenoble France; MAX IV Laboratory SE-22594 Lund Sweden; SPIN-CNR, c/o DICII-University of Rome Tor Vergata Via del Politecnico 1 I-00133 Roma Italy

## Abstract

Self-ordered porous anodic alumina (PAA) films are studied extensively due to a large number of possible applications in nanotechnology and low cost of production. Whereas empirical relationships between growth conditions and produced oxides have been established, fundamental aspects regarding pore formation and self-organization are still under debate. We present *in situ* structural studies of PAA films using grazing-incidence transmission small-angle X-ray scattering. We have considered the two most used recipes where the pores self-organize: 0.3 M H_2_SO_4_ at 25 V and 0.3 M C_2_H_2_O_4_ at 40 V. During anodization we have followed the evolution of the structural parameters: average interpore distance, length of ordered pores domains, and thickness of the porous oxide layer. Compared to the extensively used *ex situ* investigations, our approach gives an unprecedented temporal accuracy in determination of the parameters. By using of Al(100), Al(110) and Al(111) surfaces, the influence of surface orientation on the structural evolution was studied, and no significant differences in the interpore distance and domain length could be observed. However, the rate of oxide growth in 0.3 M C_2_H_2_O_4_ at 40 V was significantly influenced by the surface orientation, where the slowest growth occurs for Al(111). In 0.3 M H_2_SO_4_ at 25 V, the growth rates were higher, but the influence of surface orientation was not obvious. The structural evolution was also studied on pre-patterned aluminum surfaces. These studies show that although the initial structures of the oxides are governed by pre-patterning geometry, the final structures are dictated by the anodization conditions.

## Abstract 2

Growth of porous anodic alumina films studied *in situ* under electrochemical anodization conditions by grazing-incidence transmission small-angle X-ray scattering.

## Graphical abstract

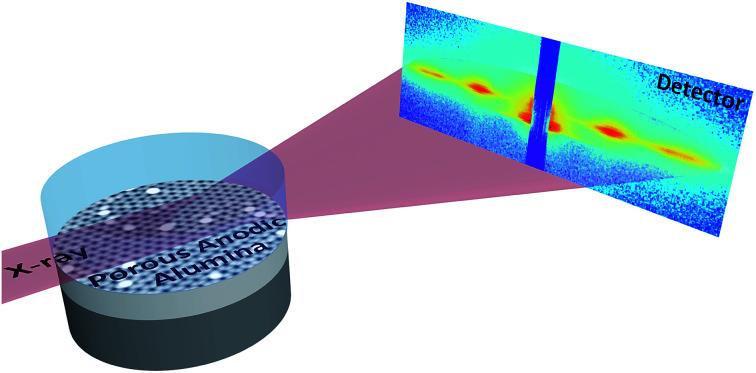

## Introduction

Aluminum and aluminum alloy materials are used in a vast number of applications due to their high strength to weight ratio, hardness and corrosion resistance.^1–3^ The corrosion resistance of Al materials can in many cases be attributed to a thin (2–7 nm) native oxide film on the material that is formed and is renewable in ambient and aqueous environments.^2,4–7^ In harsher environments such as seawater, the protection of Al parts against corrosion needs to be improved, which can be achieved by electrochemically growing a thicker oxide by anodization.^2^

During anodization, the applied anodic potential results in heterolytic dissociation of water at the oxide-electrolyte interface and an electric field generated across the growing anodic aluminum oxide (AAO) film. This electric field drives the migration of the dissociation products (O^2−^ and OH^−^ ions) towards the metal-oxide interface and Al^3+^ ions from the metal-oxide interface towards the oxide-electrolyte interface.^8^

If the anodization is performed in an electrolyte in which anodic oxide is soluble, the produced AAO will be porous.^1,2^ In this case, the migrating O^2−^/OH^−^ ions forms oxide with Al^3+^ ions at the metal-oxide interface, and the migrating Al^3+^ ions are ejected into the solution without causing oxide formation at the oxide-electrolyte interface.^8^ The porous-type oxide film consists of a compact barrier layer in contact with the aluminum and a porous layer with nanometer-sized pores on top of the barrier layer. These pores are parallel, directed perpendicularly to the sample surface and extend through the entire porous layer. Whereas the barrier layer thickness is constant and linearly related to the applied potential (about 1.1 nm V^−1^), the porous layer thickness is proportional to the ionic current, *i.e.* it is determined by the anodization time and can reach tens of μm.^9^

**Fig. 1:**
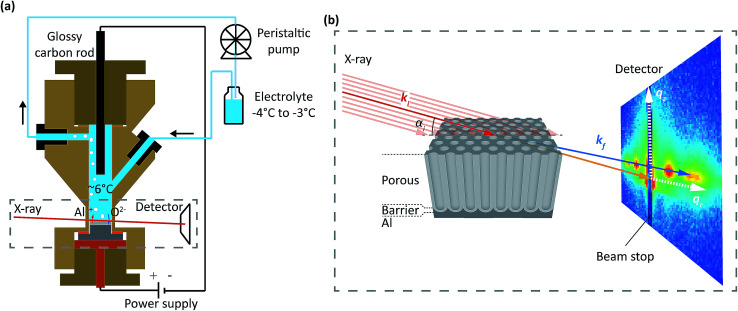
(a) Schematic of the setup with the cylindrical cell depicted in cross-section. The hat shaped sample (gray) is the working electrode (anode), and a glassy carbon rod is the counter electrode (cathode). The chilled electrolyte is continuously pumped through the cell with a peristaltic pump. Oxygen species (O^2−^) from the splitting of H_2_O migrate towards the metal-oxide interface and are involved in oxide formation. Al^3+^ from the Al substrate migrate towards the oxide-electrolyte interface, but are mostly directly ejected into the electrolyte and not involved in oxide formation. Formed gas (H_2_, O_2_) and reaction heat is removed by the electrolyte flow. (b) Schematics of the experimental geometry. The incident X-ray beam of wave vector ***k***_**i**_ (red) impinges on the sample with an incidence angle *a*_i_ towards the surface of the substrate. The intensity of the photons scattered from the sample with a wave vector ***k***_**f**_ (blue) is recorded by a 2D detector. The positions on the detector is related to the wave vector transfer ***k***_**f**_ − ***k***_**i**_ = ***q*** and the wave vector transfer coordinates *q*_*r*_ and *q*_*z*_. A beam stop was placed at *q*_*r*_ = 0 to protect the detector from high intense direct and specular reflected beams. The X-ray beam profile was always considerably larger than the oxide film thickness.

Under specific anodization conditions a self-ordering mechanism exists which propagates pore growth into more and more regular positions. If the anodization in the self-ordering regime is conducted for a sufficiently long time, the pores bottoms are hexagonally arranged with a very high degree of ordering. This arrangement is imprinted in the metal surface underneath the porous layer, and if the oxide is removed, *e.g.* by chemical etching, the metal surface becomes patterned with small concaves that repeat the positions of pore bottoms from the first anodization step. This effect has been employed to produce self-ordered porous anodic alumina (PAA) films (Fig. 1b). The ordered Al surface obtained from a first anodization step serves as a template for a second anodization step at the same conditions, hence the name two-step anodization process.^10^ The pore size of the PAA can be tailored, and pore diameters between about 10 and 400 nm have been obtained using different anodization conditions.^2,11^ These structures have successfully been used for synthesizing nanowires, nanodots and nanotubes and have been proposed for use in magnetic storage, catalysis, batteries and solar cells.^12–17^

Whereas a number of empirical relations have been established between the anodization conditions and the structural features such as interpore distance and pore size, the mechanism governing the formation, growth and self-organization of PAA is not fully known and is still actively debated. It has been argued that field-assisted oxide dissolution is the main reason for the formation of the PAA. However, recent studies have suggested different models based on the field-assisted decomposition and stress-induced viscous flow of oxide to explain pore formation and self-organization during PAA growth.^18–24^ For an improved understanding of the mechanisms, it is of importance to study the evolution *in situ* as the PAA is growing.^2^

Hard X-ray scattering methods are known to be powerful tools for investigation of material's structures and their evolution *in situ*, *e.g.* during catalytic and electrochemical processes, due to the penetrating and non-destructive nature of X-rays.^25–36^ For example, small-angle X-ray scattering (SAXS), has previously been used for *in situ* studies of PAA.^27^ In these studies, the beam was incident close to normal to the surface, and the scattered beam penetrating the entire Al material and exiting through the back of the sample was measured. However, the information on changes in the direction of beam propagation, *e.g.* the anodic oxide thickness, is difficult to obtain in such experiment geometry. If grazing-incidence small-angle X-ray scattering (GISAXS) is used, the surface sensitivity is higher, and changes in the perpendicular direction can be obtained. However, the interpretation of the data is nontrivial.^37,38^ By instead using the more recently introduced variant, grazing-incidence transmission small-angle X-ray scattering (GTSAXS) where the transmitted X-ray beam exits at the side of the sample, thicker samples can be studied, and information about changes normal to the surface can more easily be obtained since the interpretation is simpler.^38^ In addition, *in situ* studies using this method during a single anodization, quickly provides a much larger data set on structural properties than possible from *ex situ* microscopy studies. We believe that a comprehensive modelling of PAA growth could reproduce our data.

Here, we demonstrate *in situ* structural evolution studies of self-ordered PAA using GTSAXS under two different anodization conditions. We also compare the behaviours for three different surface orientations: (100), (110) and (111). Finally, we investigate the effect of initial surface pre-patterning on the structural evolution.

## Experimental and analysis procedure

### Setup and conditions

The experiments were performed at the ESRF beamline ID03 (ref. 39) using an electrochemical cell made for combined electrochemical and hard X-ray measurements at synchrotrons, similar to the cell reported in ref. 40.Fig. 1a shows an illustration of the setup with the cell depicted in cross-section. The cell is made of Polyether Ether Ketone (PEEK), is 6 cm in height and has an inner cylindrical volume of 2–3 ml. A two-electrode setup was employed, in which the hat-shaped sample was the working electrode (anode), and a glassy carbon rod was the counter electrode (cathode). The electrolyte was cooled in a chilling bath and flowed continuously through the cell using a peristaltic pump. The continuous flow removes both heat and gas produced during the anodization process. The temperature in the cell center was measured by a type K thermocouple to be between 5 °C and 7 °C.

The PAA were studied during anodization using a hard X-ray beam with a photon energy of 15 keV or 21.5 keV going through the 0.1 mm thin walls of the cell. The incident X-ray beam of wavevector ***k***_**i**_ (red) impinged on the sample with an incidence angle *α*_i_, see Fig. 1b. The transmitted and scattered X-ray beam of wavevector ***k***_**f**_ (blue) were measured with a two dimensional detector (MAXIPIX^41^) placed at a distance of 2.2 m from the sample. A frame rate of up to 0.26 Hz were used.

The positions of the scattered beam on the detector are related to the wavevector transfer ***k***_**f**_ − ***k***_**i**_ = ***q*** and the wavevector transfer coordinates *q*_*r*_ and *q*_*z*_ as illustrated on the detector in Fig. 1b.

The different conditions (electrolyte, potential and substrate surface orientation) studied in the present report are summarized in Table 1. For the four first conditions, polished aluminum substrates were anodized in the same conditions in the first and second step, *i.e.* according to the two-step anodization process.^17^ For the two last conditions, the effect of pre-patterning was evaluated, by anodizing the substrate using different conditions for the first and second steps. The anodization conditions are hereafter referred to as sulfuric acid for 0.3 M H_2_SO_4_ at 25 V DC and oxalic acid for 0.3 M C_2_H_2_O_4_ at 40 V DC.

**Table 1:** Summary of the anodization conditions and X-ray beam properties

Step	Substrate	Condition	Pretreatment	Energy (keV)	Incidence angle (°)
1^st^	Al(100), Al(110), Al(111)	0.3 M H_2_SO_4_, 25 V	Polished	15	0.3
2^nd^	Al(100), Al(110), Al(111)	0.3 M H_2_SO_4_, 25 V	Anodized in 0.3 M H_2_SO_4_, 25 V, afterwards etched	15	0.3
1^st^	Al(100), Al(111)	0.3 M C_2_H_2_O_4_, 40 V	Polished	21.5	0.3
2^nd^	Al(100), Al(110), Al(111)	0.3 M C_2_H_2_O_4_, 40 V	Anodized in 0.3 M C_2_H_2_O_4_, 40 V, afterwards etched	15	0.5
2^nd^	Al(110)	0.3 M C_2_H_2_O_4_, 40 V	Anodized in 0.3 M H_2_SO_4_ at 25 V, afterwards etched	15	0.3
2^nd^	Al(110)	0.3 M H_2_SO_4_, 25 V	Anodized in 0.3 M C_2_H_2_O_4_ at 40 V, afterwards etched	15	0.5

5% of ethanol was added to the electrolytes to avoid the electrolyte freezing in the chiller bath. The single crystal aluminum substrates were purchased from Surface Preparation Laboratory (SPL), Zaandam, The Netherlands. They had a purity of 99.9999%, and the top surface was mechanically polished with an accuracy of at least 0.1° to one of the single crystal planes and a roughness ≤0.03 μm. Between the anodization steps, the oxide films were etched by immersing the anodized substrates in a solution consisting of 0.2 M CrO_3_ and 0.6 M H_3_PO_4_ that preferentially dissolves aluminum oxide without significantly affecting the metallic aluminum underneath.

*Ex situ* topographical images were measured with a NanoWizard® II AFM (JPK Instruments AG, Germany) in tapping mode using Si cantilevers with Al coated tips. For the sulfuric acid anodized PAA a scan rate of 0.2–0.3 Hz and cantilever drive frequency of 300 kHz were used. For the oxalic acid anodized PAA a scan rate of 0.5–0.9 Hz and cantilever frequency of 296–334 kHz were used.

### Analysis procedure and extraction of growth parameters

**Fig. 2:**
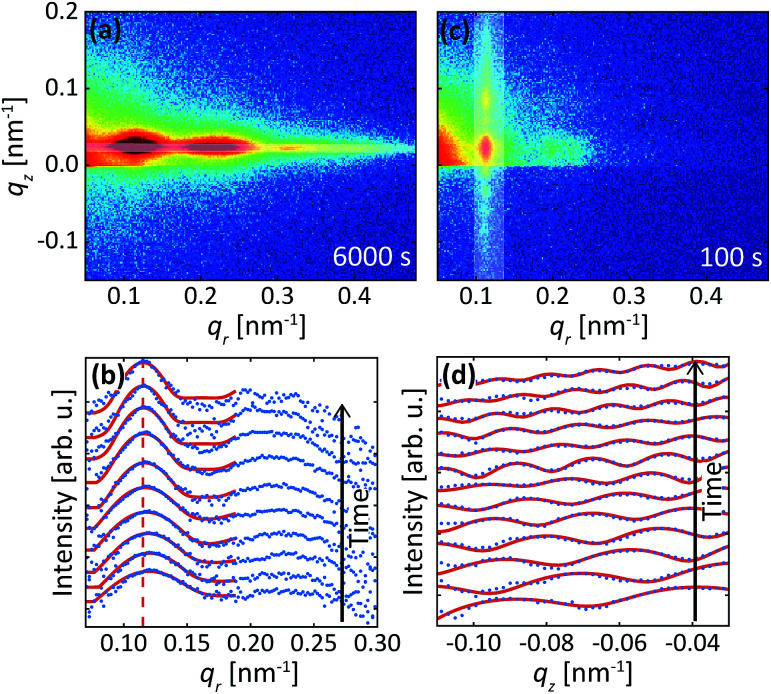
Detector image during (a) the 1^st^ anodization step and (c) 2^nd^ anodization step in 0.3 M H_2_SO_4_ at 25 V for Al(110). (b) Difference between line profiles in the *q*_*r*_ direction (blue) and fits (red). The profiles were extracted from area in the images as illustrated by the white transparent region in (a). (d) Line profiles extracted in the *q*_*z*_ direction from the images as illustrated by white transparent region in (c) and fits (red) to the profiles. A smoothing filter was applied on the profiles in (d) for visualization purposes.

The data in Fig. 2 were acquired during anodization of Al(110) in sulfuric acid and are shown for illustration of the analysis procedure. Intensity profiles in the *q*_*r*_ and *q*_*z*_ directions were extracted from the detector images, as illustrated by the white transparent regions in Fig. 2a and c, respectively. The profiles in the *q*_*r*_ direction were normalized against the background at high *q*_*r*_ where there are no intense diffraction spots. The extracted profiles correspond to the mean of the entire PAA where both oxide grown in the beginning of the anodization and newly formed oxide at the metal-oxide interface contributes to the intensity in the profiles, as the beam profile is larger than the thickness of the oxide.

Instead of analyzing the extracted profiles, the difference between the profiles in the *q*_*r*_ direction at different times, as shown in Fig. 2b, was analyzed. Under the hypothesis that the diameter of the pores do not change with time (it is already know that the pore diameter is mainly defined by the applied voltage) the change of the GTSAXS pattern between two different times is dominated by the structural correlation of the newly formed oxide layer. This procedure is similar to the isomorphic substitution procedure used in the case of X-ray diffraction from liquids or amorphous samples or to the isotopic substitution used in the case of neutrons. The reason for the use of this procedure is that the change of the GTSAXS pattern between two different times approximately corresponds to the newly formed oxide layer at the metal-oxide interface during this time. The difference *S*_T_ was calculated using the following formula1
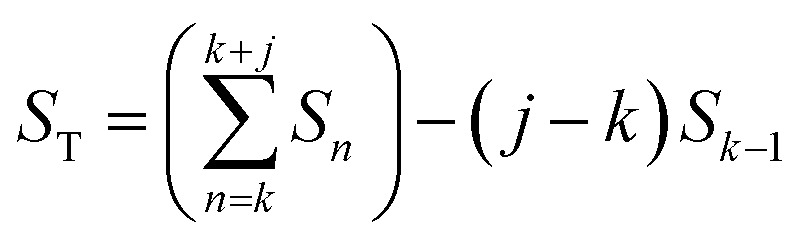
where *S*_*n*_ is the profile at time *n* and *j* is the number of images summarized after time *k*. The formula sums image *k* to *k* + *j* and subtracts this with image *S*_*k*−1_ multiplied with the same amount of images as was summed. Since the contribution from the newly formed oxide layer compared to the contribution from the entire oxide in the scattering pattern decreases as the oxide grows thicker, *j* was increased with time to obtain *S*_T_ which is statistically significant during the entire anodization time. The parameter *j* was calculated using the following formula2
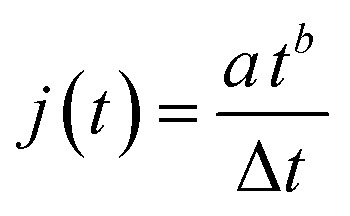
where *t* is the anodization time, Δ*t* is the time difference between two following images, and *a*, *b* are two constants describing how *j* increases with time. Several different constants *a* and *b* were tested to ensure the overall result obtained was not significantly influenced. For the anodization in sulfuric acid *a* = 6.5 and *b* = 0.65, while for anodization in oxalic acid *a* = 4 and *b* = 0.55. This approach will be discussed in greater detail in forthcoming publications.^42^

The difference profiles were fitted using the same model as we described in ref. 42 and 43. Simplified, the model is a combination of a hexagonal structure factor describing the contribution from the position of the pores and a form factor describing the contribution from the pore shape. The form factor was approximated with a hollow-shell cylinder to account for the anions incorporated in the outer region of the pore walls. In the case of anodization in sulfuric acid, a fixed inner radius of 12 nm and outer radius of 25 nm was used. In the case of anodization in oxalic acid, the inner and outer cylinder radii were fixed at 20 nm and 36 nm, respectively. The fitted momentum transfers were limited to *q*_*r*_ = 0.35 nm^−1^ and *q*_*r*_ = 0.19 nm^−1^ for the sulfuric and oxalic acid anodization, respectively. The setting of the other parameters can be found in ref. 43. The fits are shown as red lines in Fig. 2b. The interpore distance *a* was obtained from the hexagonal structure factor peak positions according to the formula3
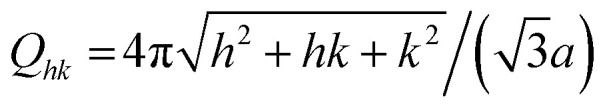
where *Q*_*hk*_ is peak positions and *h*, *k* are the Miller indices. The domain length *D* was extracted from the structure factor peak width using the formula4
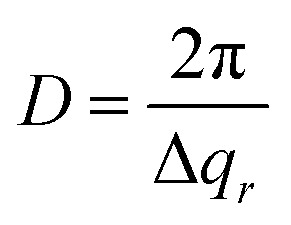
where Δ*q*_*r*_ is the peak width.

Fig. 2d shows profiles (blue dots) extracted along the *q*_*z*_ direction during the second anodization step. From the period of the observed oscillations in the *q*_*z*_ direction, the thickness *H* was determined using the formula5
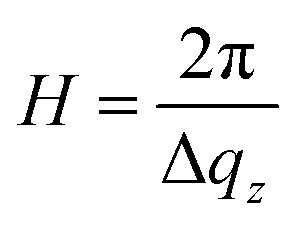
where Δ*q*_*z*_ is the period between adjacent peaks in the oscillations. The period Δ*q*_*z*_ of oscillations was determined by fitting (red line) a sinusoidal function with an exponential decreasing amplitude using a second-degree polynomial as a background.

## Results

The results are presented in six sections. In the first four sections we discuss the results obtained from the studies of the two-step anodization process using sulfuric and oxalic acid. In the two final sections we describe the results obtained using different anodization protocols for the first and the second anodization steps to evaluate the effect of surface pre-patterning.

### Two-step anodization process in sulfuric acid: first step

**Fig. 3:**
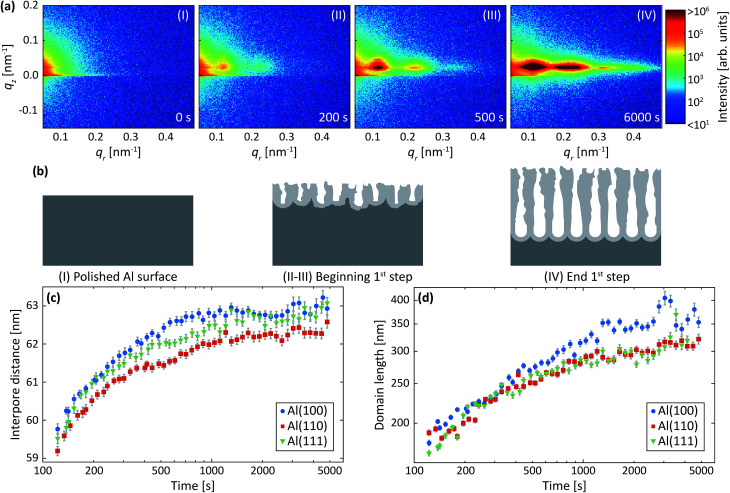
(a) Detector images recorded after (I) 0 s, (II) 200 s, (III) 500 s and (IV) 6000 s of anodization during the 1^st^ step in 0.3 M H_2_SO_4_ at 25 V for Al(110). (b) Models of the evolution of the PAA structure during the anodization. Interpore distances (c) and domain lengths (d) obtained from fits of the difference profiles in the *q*_*r*_ direction. Overlapping data points are omitted.

Fig. 3a shows detector images recorded during the first anodization step for Al(110) in sulfuric acid and in Fig. 3b an illustration of the PAA structure evolution during the anodization is depicted. The evolution of the scattering pattern can be seen in the ESI movie M1.† Before the initiation of the anodization (Fig. 3a-I) mainly diffuse scattering is visible, which is to be expected from a polished surface with little roughness.

A GTSAXS pattern with clear maxima and minima emerges after ∼200 s of anodization (Fig. 3a-II) with a diffraction spot at about *q*_*r*_ = 0.12 nm^−1^, directly indicating the existence of pores with a mean interpore distance of about 60 nm in the oxide. As the anodization progresses (Fig. 3a-(III and IV)), more diffraction spots appear in the GTSAXS pattern, and their intensities increase with time. This is consistent with an increasing volume of ordered porous oxide that diffracts the X-ray beam as the PAA increases in thickness. Fig. 3c and d shows the evolution of the average interpore distance and domain length, respectively, from the fitting of the difference profiles for Al(100), Al(110) and Al(111) obtained during the anodization in sulfuric acid. It can be seen that there is no significant difference between the different crystallographic orientations of the anodized surfaces. The interpore distance increases with time, but the change slows rapidly as a certain distance is reached. The pore formation process likely begins at imperfections^44^ with the formation of smaller and randomly positioned incipient pores with shorter interpore distances.^8,18^ As the anodization progresses, some of these incipient pores increase in size and other terminate their growth.^19,27,45^ This process continues until a stable pore size, and interpore distance is reached. Also, the average domain length increases from about 200 nm to 400 nm where the rate slows down with anodization time. To about 1000 s of anodization, the domain length increases according to a power law, which is consistent with previous observations.^45^ We find, however, that after about 15 minutes of oxidation (∼1000 s) there is an inflection in the rate of the domain length increase. The ordered domains still grow with time but at a slower rate, probably approaching their asymptotic values for these particular anodization conditions.

### Two-step anodization process in sulfuric acid: second step

**Fig. 4:**
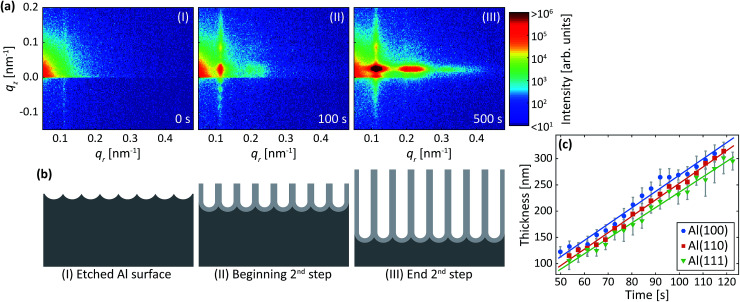
(a) Detector images recorded after (I) 0 s, (II) 100 s, (III) 500 s of anodization during the 2^nd^ step in 0.3 M H_2_SO_4_ at 25 V for Al(110). (b) Models of the evolution of the PAA structure during the anodization. (c) The thickness of the growing PAA as obtained from the period of oscillations in the *q*_*z*_ direction. The growth rates obtained from linear fits (solid lines) to the thicknesses are 3.1 nm s^−1^ (Al(100)), 3.1 nm s^−1^ (Al(110)) and 2.9 nm s^−1^ (Al(111)).

Fig. 4a shows detector images recorded during the second anodization step for Al(110) in sulfuric acid and Fig. 4b models of the evolution of the PAA structure during the anodization. The evolution of the scattering pattern can be seen in the ESI movie M2.† Before the initiation of the anodization, a streak of scattered intensity is observed at about *q*_*r*_ = 0.12 nm^−1^, see Fig. 4a-I. This streak, which does not exist before the first anodization step (Fig. 3a-I), is due to hexagonally arranged concaves with mutual separation of about 60 nm. These concaves correspond to the final positions of the pore bottoms formed in the Al during the first step, which remain imprinted in the aluminum surface after the etching.

Upon anodization, the intensity at the position of the streak increases and in addition, intensity oscillations in the *q*_*z*_ direction arise (Fig. 4a-II). The period of these oscillations decrease with anodization time, indicating an increasing PAA thickness, and after about 150 s the period becomes too small to be resolved using our experimental set-up. However, the intensity of the GTSAXS pattern further increases with time as the ordered PAA grows thicker and contributes to the intensity of the scattering pattern (Fig. 4a-III). The GTSAXS patterns are in general less diffuse during the second step than during the first step (Fig. 3a), since the pores are well ordered throughout the entire PAA instead of having high order mainly at the bottom of the pores as is the case during the first step. There is no considerable domain length evolution in the second anodization step, since the pores initiate at the pre-ordered concaves positions. This guided pore initiation have been proposed to occur due to a thinner native oxide at the bottom of the concaves.^45^

Fig. 4c shows the thicknesses of the PAA obtained from the period of the intensity oscillations in the *q*_*z*_ direction. The growth rates differs slightly for all orientations of the samples, but the difference is within the experimental error. The growth rates obtained is 2.9 nm s^−1^ for Al(111) and about 3.1 nm s^−1^ for Al(100) and Al(110).

**Fig. 5:**
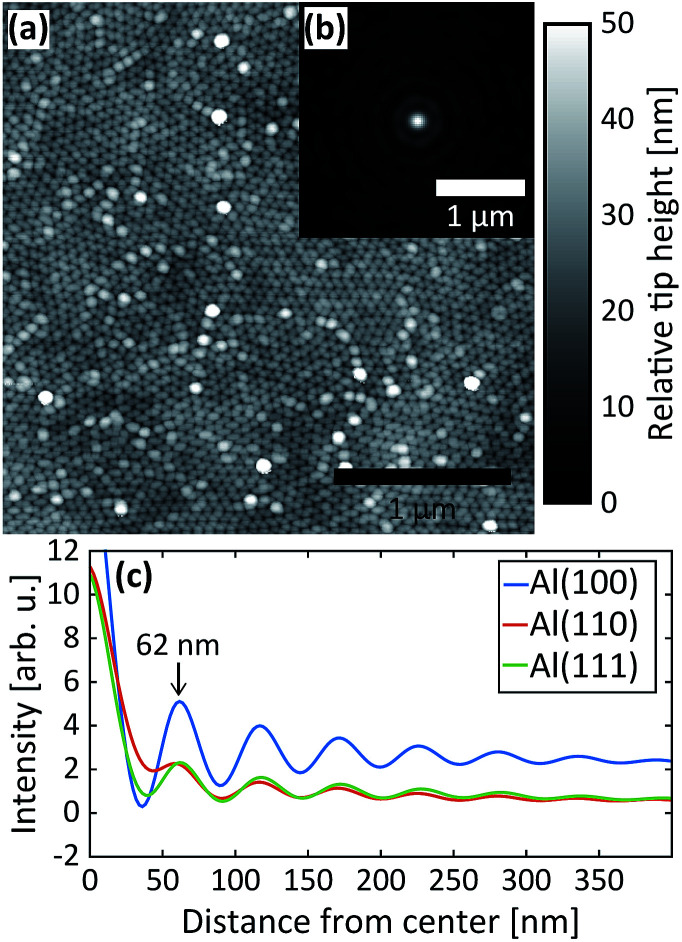
(a) AFM image of the PAA obtained after the 2^nd^ anodization step in 0.3 M H_2_SO_4_ at 25 V for Al(110). (b) The autocorrelation from the AFM image in (a) and (c) radial profiles of the autocorrelations obtained after the 2^nd^ anodization step in 0.3 M H_2_SO_4_ at 25 V for Al(100), Al(110) and Al(111).

After the second step, AFM images were measured of the PAA surfaces. The AFM image in Fig. 5a was recorded from PAA grown on the Al(110) substrate. It clearly shows the hexagonal arrangement of the pores as well as the domains. The inset (b) is the autocorrelation function of the AFM image and panel (c) plots the radial profiles of the autocorrelation function for all three surface orientations. The interpore distances obtained from the radial profiles of the autocorrelation function are about 62 nm for all orientations, which is in excellent agreement with the GTSAXS data.

### Two-step anodization process in oxalic acid: first step

**Fig. 6:**
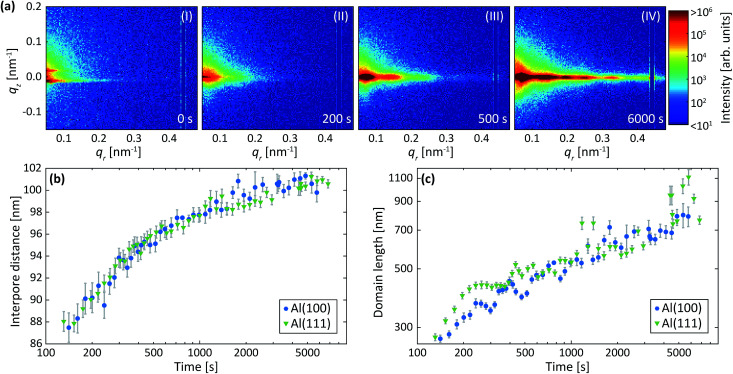
(a) Detector images recorded after (I) 0 s, (II) 200 s, (III) 500 s and (IV) 6000 s of anodization during the 1^st^ anodization step in 0.3 M C_2_H_2_O_4_ at 40 V for Al(100). (b) Interpore distances and (c) domain lengths obtained by fits of the difference profiles in the *q*_*r*_ direction as described in Section 2.2. Overlapping data points are omitted.

Fig. 6a shows detector images recorded during the first anodization step for Al(100) in oxalic acid. The development of the GTSAXS pattern during the anodization is similar to the first step in sulfuric acid as described above, showing a similar evolution of the PAA growth. However, in the present case, the diffraction peaks evolve at lower *q*_*r*_ values than in the sulfuric acid at 25 V, which is a signature of larger interpore distance. This is consistent with the expectations as the anodization potential is higher, *i.e.* 40 V.^11^

Fig. 6b and c shows the evolution of the average interpore distance and domain length from the fitting of the difference profiles for Al(100) and Al(111) obtained during the first step. Again, we find no significant difference in the growth evolution between the different surface orientations. The evolution of the interpore distance is similar to that for anodization in sulfuric acid but levels out at a higher interpore distance due to the higher anodization potential used. The rate that the domain length increases and the value of the domain length are higher for anodization in oxalic acid than in sulfuric acid.

### Two-step anodization process in oxalic acid: second step

**Fig. 7:**
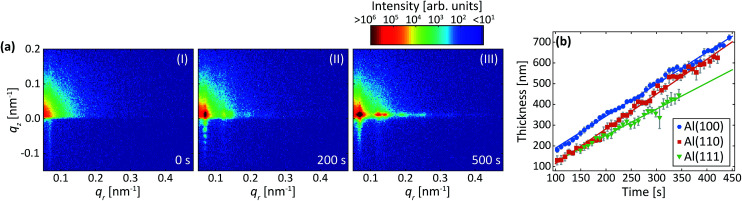
(a) Detector images recorded after (I) 0 s, (II) 200 s, (III) 500 s of anodization during the 2^nd^ step in C_2_H_2_O_4_ at 40 V for Al(100). (b) Thicknesses obtained from the period of oscillations in the *q*_*z*_ direction. The growth rates obtained from linear fits (solid lines) to the thicknesses are 1.5 nm s^−1^ (Al(100)), 1.7 nm s^−1^ (Al(110)) and 1.3 nm s^−1^ (Al(111)). Overlapping data points are omitted.

Fig. 7a are detector images recorded during the second anodization step in oxalic acid for Al(100). Again, an intensity streak due to the hexagonally arranged concaves left in the aluminum after the removal of the PAA from the first step is observed in the image (Fig. 7a-I). Now this streak is found at about *q*_*r*_ = 0.07 nm^−1^ and corresponds to a mutual separation of about 100 nm. After the onset of the second anodization step, the GTSAXS patterns evolves in a similar manner (Fig. 7a-(II and III)) as during the second anodization step in sulfuric acid.

Fig. 7b shows the evolution of the increasing thickness of the PAA obtained from the intensity oscillations observed in the *q*_*z*_ direction. The growth rates for Al(100), Al(110), and Al(111), were found to be 1.5 nm s^−1^, 1.7 nm s^−1^, and 1.3 nm s^−1^, respectively. The estimated growth rates are lower than in sulfuric acid. The slower growth rates in oxalic acid could be explained by different amounts of incorporated anions from the electrolyte, which influences the electric field profile in the PAA and in turn the ionic current and the growth rates.^46^ It could also be explained by the higher pH of the oxalic acid that is slower to dissolve the alumina of the barrier layer below the pores. The lower electric field strength across the thicker barrier layer would then lead to slower ionic transport and growth rates. Again, no statistically significant evolution of the interpore distance and domain length was observed during this step for any crystallographic orientation for the time of our experiment.

**Fig. 8:**
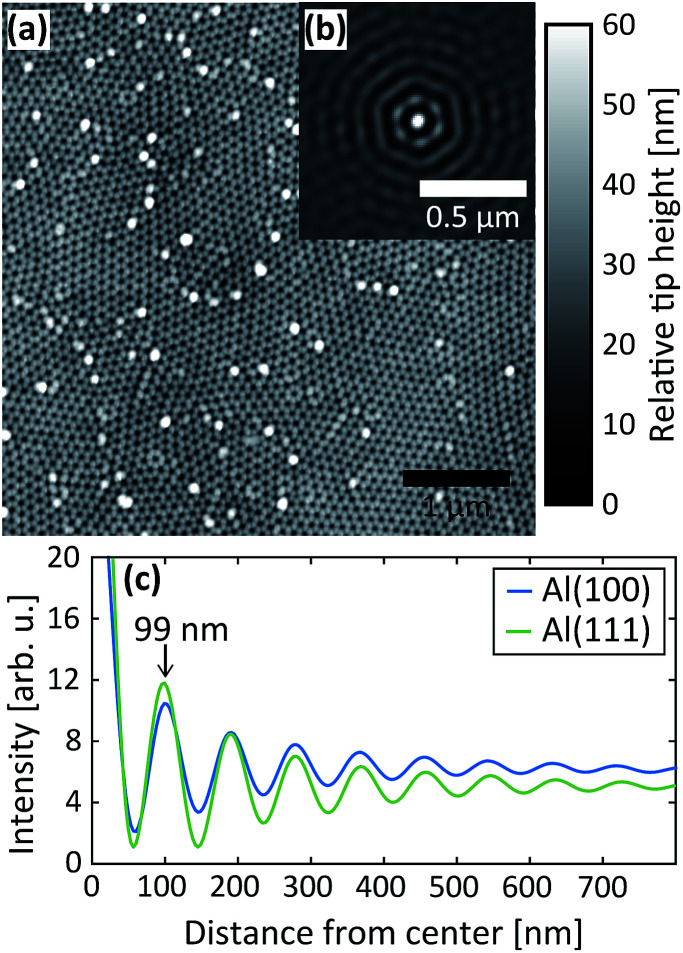
(a) AFM image of the oxide obtained after the 2^nd^ anodization step in 0.3 M C_2_H_2_O_4_ at 40 V for Al(100). (b) Autocorrelation of the AFM image in (a) and (c) radial profiles of the autocorrelations obtained after the 2^nd^ anodization step in C_2_H_2_O_4_ at 40 V for Al(100) and Al(111).

After the second step, AFM images were measured of the PAA surfaces, see Fig. 8. In Fig. 8a, an AFM image of PAA grown on Al(100) is shown, in (b) is the corresponding autocorrelation function and (c) shows the radial profile of the autocorrelation functions for the Al(100) and Al(111) surface orientations. The interpore distance obtained from the radial profile of the autocorrelation is about 99 nm for both orientations, which are in excellent agreement with the distance obtained from the GTSAXS data.

### Effect of surface pre-patterning: first anodization step in sulfuric acid at 25 V and second anodization step in oxalic acid at 40 V

To further extend our knowledge of the evolution of the anodic alumina films, we have studied the effect of the surface pre-patterning on the structural parameters of the anodic film. First, we have investigated the transition from concaves at smaller mutual separation towards pores at bigger interpore distance. To do so, we have performed the first anodization step in sulfuric acid at 25 V, followed by the etching procedure. This left us with a sample surface pre-patterned with periodically ordered concaves with a mutual separation of about 60 nm. However we performed the second anodization step using oxalic acid at 40 V, which if done with a polished sample, should result in periodically ordered pores of about 100 nm interpore distance.

**Fig. 9:**
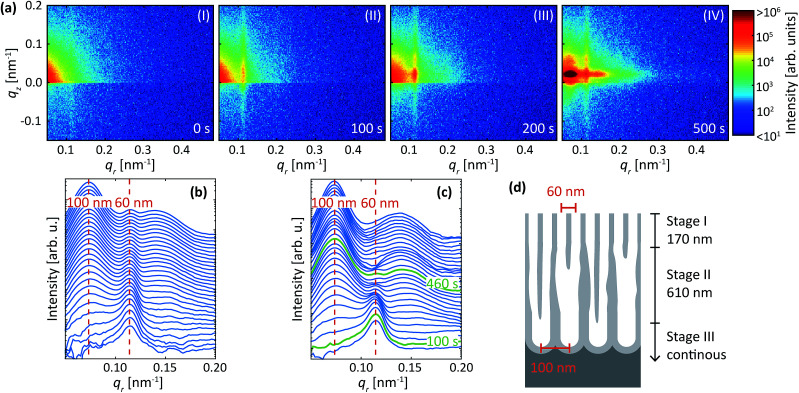
(a) Detector images recorded after (I) 0 s, (II) 100 s, (III) 200 s and (IV) 500 s during anodization in C_2_H_2_O_4_ at 40 V for Al(110) that previously been anodized in 0.3 M H_2_SO_4_ at 25 V and etched. (b) Profiles extracted in the *q*_*r*_ direction. (c) Difference profiles in the *q*_*r*_ direction. A smoothing filter was applied on the profiles for visualization purposes. The red dashed lines indicate the positions that correspond to PAA growth with either 60 nm or 100 nm interpore distance. (d) Illustration of the PAA after anodization with growth stages indicated. The green profiles in (c) indicate approximately where the growth rate is switching from predominantly 60 nm and to predominantly 100 nm interpore distance.

Fig. 9a are detector images recorded during the second step (*i.e.* in oxalic acid at 40 V) of the protocol described above for an Al(110) surface. Prior to the anodization in oxalic acid (Fig. 9a-I), a streak along *q*_*z*_ = 0.012 nm^−1^ is visible due to the concaves with a mutual separation of about 60 nm. After the initiation of the anodization (Fig. 9a-II), the intensity increases mainly at *q*_*r*_ = 0.012 nm, indicating the growth of pores with interpore distance of about 60 nm, and not an interpore distance of about 100 nm as would be the expected distance for oxalic acid at 40 V. This confirms a strong influence of the surface morphology on the initial structure of the porous oxide films, which is in agreement with the results presented above when the same condition as used for the first step was also used for the second step. As the anodization progresses (Fig. 9a-III), the diffraction spot at *q*_*r*_ = 0.012 nm^−1^ continues to increase, but in addition, a new diffraction spot appears at *q*_*r*_ = 0.07 nm^−1^. This spot arises from a PAA with interpore distance of 100 nm, which is the expected value for 40 V in oxalic acid. As the anodization continues the intensity of the spot at *q*_*r*_ = 0.012 nm^−1^ becomes constant and mainly the spot at *q*_*r*_ = 0.07 nm^−1^ increases, indicating that the PAA at this stage growths with pores having an interpore distance of 100 nm (Fig. 9a-IV).

The evolution of the PAA can also be followed from the extracted profiles and difference between the profiles as shown in Fig. 9b and c, respectively. The difference between the profiles is in this section calculated by averaging 10 profiles around time *t* and subtracting the average of 10 profiles around *t* − 80 s. From the difference profiles it is clear that only the intensity of the 60 nm peak increases in the first 100 s. Beyond 100 s of anodization, the increase of the 60 nm peak slows down, and the peak at 100 nm begins to increase. After 460 s of anodization, the signal at 60 nm remains constant. The intensity of the diffraction peak at 100 nm further increases. A model of the growth process is shown in Fig. 9d, which is made using the times shown in Fig. 9c and by assuming that the growth rate of the PAA on Al(110) is 1.7 nm s^−1^. The process is explained by three stages (I–III), in which PAA grow with interpore distance of 60 nm the first 170 nm (stage I), re-ordering occurs towards oxide growths with 100 nm interpore distance (stage II), and steady state growth occurs with 100 nm interpore distance (stage III). It is clear that the details of the conclusion and on-set of the two different PAA growth would not have been observed without studying the difference between profiles.

### Effect of surface pre-patterning: first anodization step in oxalic acid at 40 V and second anodization step in sulfuric acid at 25 V

Further, we have investigated the reverse of the protocol described above. Namely, we have performed the first anodization step with oxalic acid at 40 V, which, combined with etching, resulted in nano concaves with about 100 nm mutual separation. This was followed by anodization in sulfuric acid at 25 V, which results for polished sample in the formation of self-ordered porous structure with approximately 60 nm interpore distance.

**Fig. 10:**
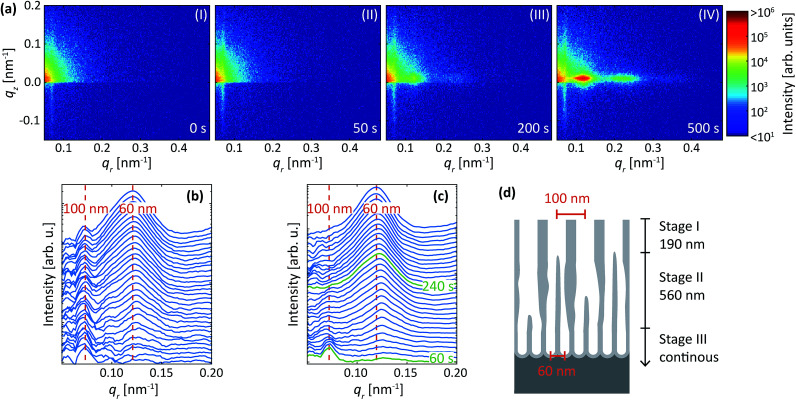
(a) Detector images recorded after (I) 0 s, (II) 50 s, (III) 200 s and (IV) 500 s of anodization during anodization in 0.3 M H_2_SO_4_ at 25 V for Al(110) that previously had been anodized in 0.3 M C_2_H_2_O_4_ at 40 V and etched. (b) Profiles extracted in the *q*_*r*_ direction. (c) Difference profiles in the *q*_*r*_ direction. A smoothing filter was applied on the profiles for visualization purposes. The red dashed lines indicate the positions that correspond to PAA growth with either 60 nm or 100 nm interpore distance. (d) Schematics of the PAA after anodization with growth stages indicated. The green profiles in (c) indicate approximately where the growth rate is switching from predominantly 100 nm and to predominantly 60 nm interpore distance.

Fig. 10a shows detector images during the second (*i.e.* with sulfuric acid at 25 V) anodization step of this reversed protocol for Al(110) surface. Before the anodization in sulfuric acid (Fig. 10a-I), an intensity streak along *q*_*r*_ = 0.07 nm^−1^ is visible due to the concaves with a mutual separation of about 100 nm. Shortly after the initiation of the anodization in sulfuric acid at 25 V, the intensity increase was detected mainly at *q*_*r*_ = 0.07 nm^−1^, indicating the growth of pores with interpore distance of about 100 nm (Fig. 10a-II). This again confirms the importance of the surface pre-patterning for the initial morphology of the anodic films.

Upon the second anodization step (Fig. 10a-III), the diffraction spot at *q*_*r*_ = 0.07 nm^−1^ stops increasing in intensity, but instead, a new spot emerges at *q*_*r*_ = 0.12 nm^−1^. This spot corresponds to a (10) diffraction signal from a structure with interpore distance of about 60 nm, which is expected for the used condition. As the anodization progresses, the intensity of the spot at *q*_*r*_ = 0.12 nm^−1^ increases in intensity, indicating that the PAA now grows with only an interpore distance of 60 nm (Fig. 10a-IV).

The profiles and difference profiles are shown in Fig. 10b and c, respectively. The difference profiles were calculated using the same procedure as described above. Again, from the difference profiles, it is clear that only the intensity of the 100 nm peak increases until about 60 s. After this time, the rate at which the peak increases slows down, and the intensity of the 60 nm peak begins to increase instead. After 240 s, the signal at 100 nm becomes constant and only the 60 nm peak increases in intensity. A model of the growth process is shown in Fig. 10d, constructed by using the times shown in Fig. 10c and by assuming that the growth rate of Al(110) is 3.1 nm s^−1^. The process is explained by three stages (I-III), where PAA with an interpore distance of 100 nm dominates the growth at the beginning of the anodization (stage I), while re-ordering occurs towards PAA growth with 60 nm interpore distance (stage II), and a steady state growth occurs with 60 nm interpore distance (stage III).

## Discussion

The structural details of an electrode interface under operational conditions are difficult to obtain due to the presence of the electrolyte. The structure is therefore often studied *ex situ*, before and after the electrode has been in operation,^45^ limiting the number of structural data points simply by the time-consuming aspect of such experiments. There has been an effort over many years to perform *in situ* measurements. The goal was to reveal structural transitions as the electrode works and to combine this information with measurements of various other phenomena such as catalytic conversion of organic species or electrodeposition of metals. In applied electrochemistry, information on the electrode surface structure and composition can be studied by *in situ* electrochemical techniques such as cyclic voltammetry (CV)^47^ and electrochemical impedance spectroscopy (EIS)^28,30,48–50^ which provide indirect structural information *via* modelling the electronic properties of the electrode/electrolyte materials system. For more fundamental studies, various electrochemical scanning probe microscopy techniques (EC-AFM, EC-STM, SKPFM)^49,51^ have during the last 20–30 years, provided a significant contribution to the understanding of electrode reconstructions^52^ and localized corrosion phenomena.^53^ Very recently, electrochemical X-ray photoemission spectroscopy (E-XPS) has become available thanks to the development of differentially pumped electron analyzer designs and suitable electrochemical environments.^54^

The use of hard X-rays (10–100 keV) for *in situ* studies of electrodes was recognized early as a valuable tool.^40,55,56^ In particular, the ability to penetrate through the electrolyte and scatter from the electrode surface is of significant importance. To this end, a number of *in situ* hard X-ray studies on electrodes and electrode surfaces has been performed previously using techniques such as X-ray reflectivity (XRR),^28–30^ surface X-ray diffraction (SXRD),^34,36,56^ transmission surface diffraction (TSD)^57^ and more. Small angle X-ray scattering (SAXS) has also successfully been used in an electrochemical environment,^27^ to probe the structure of PAA. We have previously shown that grazing-incidence transmission small-angle X-ray scattering (GTSAXS) is also applicable to study the long-range structural evolution of an electrode under harsh electrochemical environments,^43^ with the benefit that modelling of the data is much simpler as compared to the data obtained using GISAXS.

In particular, by the use of an intense X-ray beam from a synchrotron, it is possible to disentangle the structural evolution in the electrochemical environment by the difference procedure outlined in the present report. For instance, we can show that although the electrolyte and potential are optimized for a PAA with a specific interpore distance, the initial PAA interpore distance will be determined by the previously pre-patterned surface. In fact, by using this approach, we can determine at which thickness the PAA is changing to the expected interpore distance. This observation would have been obscured without the subtraction approach, which will be described in more detail in a forthcoming publication.^42^ Another unique property of the present GTSAXS measurements is that they provide an accurate direct, *in situ*, estimate for growth rates at the early stages of the second anodization step.

The use of GTSAXS for the study of PAA as in the present report opens the door for nearly unlimited investigations of the formation and functionalization of PAA. Moreover, this approach is not limited to studying PAA exclusively, but may be applied to derive properties of other hierarchically-ordered materials. For electrodeposition, the barrier layer thickness below the pores needs to be decreased by an etching approach either by increasing the electrolyte temperature or by stepping down the anodization potential,^58^ a process that could be observed *in situ*. The subsequent electrodeposition could probably then be followed *in situ*, in particular when using anomalous GTSAXS. Using a nano-sized X-ray beam, the electrodeposited material can then be mapped inside the pores and combining the GTSAXS with X-ray fluorescence and X-ray absorption near edge structure (XANES) the chemical state of the electrodeposited material can be determined. These properties may be crucial for a functional device such as a catalyst or a solar cell.

## Summary and conclusions

In this report, GTSAXS has been used for studies of the two-step anodization process of aluminum single crystal surfaces in 0.3 M H_2_SO_4_ at 25 V and 0.3 M C_2_H_2_O_4_ at 40 V. During the first step for both conditions the evolution of the interpore distance was followed and a maximum interpore distance of about 60 nm and 100 nm were reached in sulfuric acid and oxalic acid, respectively. These values are in an excellent agreement with the previously reported empirical law, where the interpore distance is related to the anodization potential as 2.5 nm V^−1^. The domain lengths increased approximately according to a power law. The increase and the final domain length obtained after 2 h anodization were higher for samples anodized in oxalic acid.

The thicknesses of the growing PAA films could be determined during the second anodization step. The growth rate was found to be higher for sulfuric acid than for oxalic acid. For both conditions the surface orientation affected the growth rates, demonstrating in particular that the (111) displays a slower growth rate. However, while the impact of the surface orientation on the porous oxide growth for oxalic acid is pronounced, for sulfuric acid it is more a trend on the border of statistical significance.

The effect of surface pre-patterning on the self-ordering was also studied by using different anodization conditions in the first and the second anodization steps. The results showed that at the beginning of the second anodization step the pores followed the morphology inherited from the previous step no matter the anodization conditions. However, further anodization resulted in re-ordering of the pores and PAA growth according to the optimal interpore distance for the given anodization conditions.

To conclude, the reports demonstrate the unique opportunity GTSAXS provides for time-resolved *in situ* studies of the morphology and structure during growth of self-ordered PAA. The possibility of using GTSAXS, for morphology determination, in combination with methods that can control ordering parameters such as stress, could be an important tool for the understanding of the mechanism behind the pore formation and self-organization. Further, the use of GTSAXS is not limited to studies of self-organization, but the use can be extended to studies of nanostructure synthesis in the pores and functional nanodevices based on the PAA.

## Conflicts of interest

There are no conflicts to declare.

## Supplementary Material

RA-008-C8RA02913J-s001

RA-008-C8RA02913J-s002

## References

[cit1] Diggle J. W., Downie T. C., Goulding C. W. (1969). Chem. Rev..

[cit2] Lee W., Park S.-J. (2014). Chem. Rev..

[cit3] Thompson G. E., Habazaki H., Shimizu K., Sakairi M., Skeldon P., Zhou X., Wood G. C. (1999). Aircr. Eng..

[cit4] Evertsson J., Bertram F., Zhang F., Rullik L., Merte L. R., Shipilin M., Soldemo M., Ahmadi S., Vinogradov N., Carlà F., Weissenrieder J., Göthelid M., Pan J., Mikkelsen A., Nilsson J. O., Lundgren E. (2015). Appl. Surf. Sci..

[cit5] Cabrera N., Mott N. F. (1948). Rep. Prog. Phys..

[cit6] Fehlner F. P., Mott N. F. (1970). Oxid. Met..

[cit7] Schultze J. W., Lohrengel M. M. (2000). Electrochim. Acta.

[cit8] Thompson G. E. (1997). Thin Solid Films.

[cit9] Wernick S. , Pinner R. and Sheasby P. G., The surface treatment and finishing of aluminium and its alloys, ASM International, Ohio, 5th edn, 1987

[cit10] Masuda H., Fukuda K. (1995). Science.

[cit11] Li A. P., Müller F., Birner A., Nielsch K., Gösele U. (1998). J. Appl. Phys..

[cit12] Berganza E., Bran C., Jaafar M., Vázquez M., Asenjo A. (2016). Sci. Rep..

[cit13] Pellin M. J., Stair P. C., Xiong G., Elam J. W., Birrell J., Curtiss L., George S. M., Han C. Y., Iton L., Kung H., Kung M., Wang H.-H. (2005). Catal. Lett..

[cit14] Gowda S. R., Leela Mohana Reddy A., Zhan X., Ajayan P. M. (2011). Nano Lett..

[cit15] Roy P., Berger S., Schmuki P. (2011). Angew. Chem., Int. Ed..

[cit16] Chun H., Hahm M. G., Homma Y., Meritz R., Kuramochi K., Menon L., Ci L., Ajayan P. M., Jung Y. J. (2009). ACS Nano.

[cit17] Masuda H., Satoh M. (1996). Jpn. J. Appl. Phys..

[cit18] Oh J., Thompson C. V. (2011). Electrochim. Acta.

[cit19] Baron-Wiecheć A., Burke M. G., Hashimoto T., Liu H., Skeldon P., Thompson G. E., Habazaki H., Ganem J. J., Vickridge I. C. (2013). Electrochim. Acta.

[cit20] Garcia-Vergara S. J., Skeldon P., Thompson G. E., Habazaki H. (2006). Electrochim. Acta.

[cit21] Cheng C., Ngan A. H. W. (2011). Electrochim. Acta.

[cit22] Cheng C., Ngan A. H. W. (2013). J. Phys. Chem. C.

[cit23] Houser J. E., Hebert K. R. (2009). Nat. Mater..

[cit24] Hebert K. R., Albu S. P., Paramasivam I., Schmuki P. (2012). Nat. Mater..

[cit25] Essehli R., El Bali B., Faik A., Benmokhtar S., Manoun B., Zhang Y., Zhang X. J., Zhou Z., Fuess H. (2012). J. Alloys Compd..

[cit26] Renner F. U., Stierle A., Dosch H., Kolb D. M., Lee T. L., Zegenhagen J. (2006). Nature.

[cit27] Napolskii K. S., Roslyakov I. V., Eliseev A. A., Byelov D. V., Petukhov A. V., Grigoryeva N. A., Bouwman W. G., Lukashin A. V., Chumakov A. P., Grigoriev S. V. (2011). J. Phys. Chem. C.

[cit28] Zhang F., Evertsson J., Bertram F., Rullik L., Carla F., Långberg M., Lundgren E., Pan J. (2017). Electrochim. Acta.

[cit29] Bertram F., Zhang F., Evertsson J., Carlà F., Pan J., Messing M. E., Mikkelsen A., Nilsson J.-O., Lundgren E. (2014). J. Appl. Phys..

[cit30] Evertsson J., Bertram F., Rullik L., Harlow G., Lundgren E. (2017). J. Electroanal. Chem..

[cit31] Napolskii K. S., Roslyakov I. V., Romanchuk A. Y., Kapitanova O. O., Mankevich A. S., Lebedev V. A., Eliseev A. A. (2012). J. Mater. Chem..

[cit32] Gustafson J., Shipilin M., Zhang C., Stierle A., Hejral U., Ruett U., Gutowski O., Carlsson P.-A., Skoglundh M., Lundgren E. (2014). Science.

[cit33] Stamenkovic V. R., Fowler B., Mun B. S., Wang G., Ross P. N., Lucas C. A., Marković N. M. (2007). Science.

[cit34] Lucas C. A., Cormack M., Gallagher M. E., Brownrigg A., Thompson P., Fowler B., Grunder Y., Roy J., Stamenkovic V., Markovic N. M. (2009). Faraday Discuss..

[cit35] Lucas C. A., Thompson P., Gründer Y., Markovic N. M. (2011). Electrochem. Commun..

[cit36] Gründer Y., Lucas C. A. (2016). Nano Energy.

[cit37] Renaud G., Lazzari R., Leroy F. (2009). Surf. Sci. Rep..

[cit38] Lu X., Yager K. G., Johnston D., Black C. T., Ocko B. M. (2013). J. Appl. Crystallogr..

[cit39] Ferrer S., Comin F. (1995). Rev. Sci. Instrum..

[cit40] Foresti M. L., Pozzi A., Innocenti M., Pezzatini G., Loglio F., Salvietti E., Giusti A., D'Anca F., Felici R., Borgatti F. (2006). Electrochim. Acta.

[cit41] Ponchut C., Rigal J. M., Clément J., Papillon E., Homs A., Petitdemange S. (2011). J. Instrum..

[cit42] Felici R. , Vinogradov N., Harlow G. S., Carlà F., Evertsson J., Rullik L., Linpé W. and Lundgren E., in manuscript 10.1039/c8ra02913j PMC9080605 35539633

[cit43] Vinogradov N. A., Harlow G. S., Carlà F., Evertsson J., Rullik L., Linpé W., Felici R., Lundgren E. (2018). ACS Appl. Nano Mater..

[cit44] O'Sullivan J. P., Wood G. C. (1970). Proc. R. Soc. London, Ser. A.

[cit45] Li F., Zhang L., Metzger R. M. (1998). Chem. Mater..

[cit46] Thompson G. E., Wood G. C. (1981). Nature.

[cit47] Bard A. J. and Faulkner L. R., Electrochemical Methods: Fundamentals and Applications, John Wiley & Sons, Inc, 2nd edn, 2001

[cit48] Mansfeld F., Kendig M. W. (1988). J. Electrochem. Soc..

[cit49] Zhang F., Nilsson J.-O., Pan J. (2016). J. Electrochem. Soc..

[cit50] Orazem M. E. and Tribollet B., Electrochemical Impedance Spectroscopy, John Wiley & Sons, 2008

[cit51] Yagati A. K. , Min J. and Choi J.-W., in Modern Electrochemical Methods in Nano, Surface and Corrosion Science, ed. M. Aliofkhazraei, InTech, Rijeka, ch. 03, 2014, 10.5772/57236

[cit52] Wilms M., Broekmann P., Stuhlmann C., Wandelt K. (1998). Surf. Sci..

[cit53] Davoodi A., Pan J., Leygraf C., Norgren S. (2005). Electrochem. Solid-State Lett..

[cit54] Axnanda S., Crumlin E. J., Mao B., Rani S., Chang R., Karlsson P. G., Edwards M. O. M., Lundqvist M., Moberg R., Ross P., Hussain Z., Liu Z. (2015). Sci. Rep..

[cit55] Ryan M. P., Toney M. F., Davenport A. J., Oblonsky L. J. (1999). MRS Bull..

[cit56] Fong D. D., Lucas C. A., Richard M.-I., Toney M. F. (2010). MRS Bull..

[cit57] Reikowski F., Wiegmann T., Stettner J., Drnec J., Honkimäki V., Maroun F., Allongue P., Magnussen O. M. (2017). J. Phys. Chem. Lett..

[cit58] Gerein N. J., Haber J. A. (2005). J. Phys. Chem. B.

